# ‘What is psychiatry?’ – an exploration of the effect of a psychiatry summer school on school students’ attitudes towards psychiatry, through the medium of word clouds

**DOI:** 10.1192/bjo.2021.451

**Published:** 2021-06-18

**Authors:** Clementine Wyke, Charlotte Wilson Jones, James Chivers

**Affiliations:** South London and Maudsley NHS Foundation Trust

## Abstract

**Aims:**

To explore if attending a psychiatry summer school would change the understanding of school students as to what the word ‘Psychiatry’ represents.

**Background:**

The Institute of Psychiatry, Psychology and Neuroscience (IoPPN) and the local mental health trust, South London and Maudsley NHS Foundation Trust (SLaM) ran a free five-day summer school for 16-year-old school students, who had just completed their GCSE exams, from state and private secondary schools within South-East London.

**Method:**

We asked all 26 student attendees to anonymously write down as many single words relating to ‘Psychiatry’ as they could think of. They were given approximately 5 minutes to complete this and they were asked to do this at the beginning of the first day and at the end of the final day of the summer school. These words were then transcribed with the number of times each word was submitted being documented. This information was then formatted into a word cloud, with the size of the word varying according to how many times it had been submitted.

**Result:**

At the start of the summer school, the students submitted a total of 208 words which included a total of 94 distinct words. Of these, the 2 most common were brain (n = 15) and mental (n = 10). At the end of the summer school, the students submitted a total of 199 words which included a total of 100 distinct words. The 2 most common were psychosis (n = 12) and forensic (n = 8). Of the words submitted pre-summer school, there were 8 distinct words that described positive attributes of psychiatry – such as ‘helping’. This increased to 17 distinct positive words post-summer school.

**Conclusion:**

We note from our outcomes that the number of words submitted by the students pre and post the summer school were similar but the words submitted most frequently differed. The most common words submitted post the summer school were more consistent with medical terminology than those submitted pre the summer school, which suggests that their knowledge of this had increased. The increase in the number of distinct positive words submitted at the end of the summer school implies that the students had a more positive view of psychiatry following the summer school.

